# COST-EFFECTIVENESS OF COGNITIVE BEHAVIOURAL THERAPY VERSUS HEALTH EDUCATION FOR SLEEP DISTURBANCE AND FATIGUE FOLLOWING STROKE AND TRAUMATIC BRAIN INJURY

**DOI:** 10.2340/jrm.v57.42770

**Published:** 2025-04-24

**Authors:** Duncan MORTIMER, Lucy YMER, Adam MCKAY, Dana WONG, Kate FRENCHAM, Natalie GRIMA, Monique ROPER, Sylvia NGUYEN, Jade MURRAY, Gershon SPITZ, Jennie PONSFORD

**Affiliations:** 1 Centre for Health Economics, Monash Business School, Monash University, Melbourne; 2 Monash Epworth Rehabilitation Research Centre, School of Psychological Sciences, Monash University, Melbourne; 3 Epworth Healthcare, Melbourne; 4 School of Psychology and Public Health, La Trobe University, Melbourne, VIC, Australia

**Keywords:** acquired brain injury, stroke, sleep, fatigue, cognitive behavioural therapy, health education, economic evaluation, cost-effectiveness

## Abstract

**Objective:**

Evaluate cost, effectiveness and cost-effectiveness of cognitive behavioural therapy for sleep and fatigue (CBT-SF) vs health education (HE) and of CBT-SF vs treatment as usual (TAU) for sleep disturbance and fatigue in acquired brain injury.

**Design:**

Economic evaluation from Australian health system and societal perspectives based on data from a June 2017 to October 2023 randomized controlled trial.

**Subjects:**

Community-dwelling Australian adults with sleep disturbance and fatigue following acquired brain injury (*n* = 126).

**Methods:**

Incremental health system costs based on cost of delivery and health service utilization since last follow-up. Incremental effectiveness based on participant-reported sleep quality, fatigue, and quality of life at each timepoint. Productivity gains/losses based on a 1-week activity diary at each timepoint.

**Results:**

Reductions in health service utilization from CBT-SF (–A$777, 95% CI: –A$4,232, A$2,678) offset higher delivery costs (A$333, 95% CI: A$109, A$556) relative to HE, with improvements in quality of life at 2 months post-treatment (0.02, 95% CI: –0.01, 0.05) and an additional 3.37 quality-adjusted life days per participant (95% CI: –4.18, 10.92). CBT-SF dominates HE (less costly and more effective) and is likely more cost-effective than HE (66–76%). CBT-SF is cost-effective *relative to TAU* under realistic assumptions.

**Conclusions:**

CBT-SF after acquired brain injury improved clinical and economic outcomes and was more likely to be cost-effective than HE. Further research is required to precisely estimate the cost-effectiveness of CBT-SF vs TAU and to demonstrate generalizability to routine practice and other settings.

**ANZCTR Trial registration numbers:**

1261700087830; 12617000879369.

## LAY ABSTRACT

People with acquired brain injury often suffer from sleep disturbance and fatigue. Cognitive behaviour therapy can improve sleep quality and fatigue. This study evaluated the costs and benefits of giving cognitive behaviour therapy for sleep problems and fatigue to people with acquired brain injury. It is based on data from a clinical trial in which 126 adults with traumatic brain injury or stroke received either 8 weeks of cognitive behaviour therapy or health education. The trial collected information on sleep quality, fatigue, quality of life, productivity, and health service use before treatment, after treatment, and at 2 months and 4 months after treatment. On average, sleep quality, fatigue, and quality of life were better after people received cognitive behaviour therapy than after health education. This was also true for health service use and productivity. Cognitive behaviour therapy was on average the less costly and more effective option *and* more likely to be cost-effective than health education.

Sleep disturbance and fatigue are common, persistent, and debilitating symptoms of acquired brain injury (1, 2). Among the available treatment options (3), cognitive behaviour therapy targeting sleep quality and fatigue (CBT-SF) and adapted for use in individuals with traumatic brain injury (TBI) and stroke shows particular promise (4, 5). Recent evidence (6) demonstrates significant improvements in sleep quality and fatigue from an 8-week CBT-SF intervention as compared with an active health education control. These initial impacts on sleep quality and fatigue may translate into broader clinical and economic benefits, potentially impacting rehabilitation outcomes, downstream health service utilization, economic participation, and quality of life (7, 8).

CBT has application in a broad range of mental health conditions, including depression, anxiety, and obsessive-compulsive disorder (9). CBT aims to help people identify and modify maladaptive thoughts and behaviours that contribute to mental health conditions. In this study, CBT focused on addressing both sleep disturbance and fatigue as these problems coexist in > 50% of people with acquired brain injury (ABI) (6).

The cost-effectiveness of CBT for sleep disturbance and associated fatigue has previously been demonstrated in other target populations (10). However, the aetiology and consequences of sleep disturbance and fatigue are different in ABI populations than in non-ABI populations and CBT-SF must be adapted to accommodate common cognitive impairments after ABI (11, 12). For ABI populations, the cost-effectiveness of behavioural therapy for anxiety has previously been evaluated in stroke survivors with aphasia (13) and there is a comparatively well-developed evidence base regarding the cost-effectiveness of neuropsychological interventions for rehabilitation (14). However, none of the available evidence speaks directly to the cost-effectiveness of CBT-based treatment for both sleep disturbance *and* fatigue, and the cost-effectiveness of CBT-SF has not yet been demonstrated in an ABI population. The present study aims to remedy this gap in the literature and is the first study to evaluate the cost, effectiveness, and cost-effectiveness of CBT-SF for alleviating sleep disturbance and fatigue in people with ABI.

## Methods

### Design

Trial-based economic evaluation alongside a parallel 2-group randomized controlled trial comparing manualized CBT-SF vs Health Education (HE) for sleep disturbance and fatigue following ABI (6). The trial-based economic evaluation was designed to: (*i*) describe additional resources required to deliver manualized CBT-SF as compared with HE and TAU, (*ii*) evaluate whether CBT-SF is cost-saving in comparison with HE and TAU, and (*iii*) evaluate the cost-effectiveness of CBT-SF by comparing incremental cost against improvements in clinical and economic outcomes.

Base-case analyses were conducted from the perspective of the Australian public health system, with supplementary analyses encompassing productivity gains/losses to consider the societal perspective. The time horizon for inclusion of relevant costs and economic outcomes coincides with the study period (from baseline to 4 months post-treatment) but clinical outcomes (and incremental cost-effectiveness based on clinical outcomes) were evaluated based on treatment effects at post-treatment, 2 months post-treatment, and 4-months post-treatment.

Ethical approval for this study was initially obtained from the relevant hospital Human Research Ethics Committees (RES-19-0000178E). Prospective registration was undertaken with Australian New Zealand Clinical Trials Registry for TBI (12617000878370) and stroke (12617000879369) in June 2017.

Further details regarding manualization of the intervention, design of the trial, and methods for the main effectiveness analysis are reported elsewhere (6). Further details regarding methods for the trial-based evaluation are provided below and in Appendix S1; Supplementary Tables S1–S4).

### Participants

Recruitment, randomization, and inclusion/exclusion of participants were as for the main effectiveness analysis (15). Briefly, participants were community-dwelling adults (aged 16–71) with sleep disturbance (Pittsburgh Sleep Quality Index > 5 [PSQI] [16]) and/or fatigue (Fatigue Severity Scale ≥ 4 [FSS] [17]) following stroke or TBI; recruited from ABI clinicians, stroke support groups/websites, and a longitudinal TBI research database (18). Participants (*n* = 126) were randomized in a 2:1 ratio to intervention (*n* = 86) or control (*n* = 40); costs and cost-effectiveness were therefore calculated and reported per participant.

### Intervention and comparator

Participants randomized to the intervention group received 8 weeks of manualized CBT-SF that included weekly, one-on-one sessions with a clinical neuropsychologist (15). Further details regarding delivery and resource requirements for manualized CBT-SF have been provided in Appendix S1; Supplementary Tables S1 and S4). Participants randomized to the control group received an 8-week Health Education (HE) intervention designed for the purposes of this trial (12) that included weekly one-on-one sessions of HE with a clinical neuropsychologist plus usual care. Further details regarding delivery and resource requirements for the HE intervention have been provided in Appendix S1; Supplementary Tables S2 and S4).

### Incremental effectiveness

The primary outcome for the cost-effectiveness analysis was SF6D-based quality-adjusted life days (QALDs) from baseline to final follow-up. SF6D Index Scores were derived from participant-level SF36 item response data (19) at each timepoint and standard gamble weights from a UK general population sample (20). SF36-based SF6D scores range between 0.350 (“the pits”) and 1.000 (“full health”); higher scores indicate higher health-related quality of life (20). By contrast, quality of life was measured in the main effectiveness analysis (6) using SF36 Mental Component Summary (MCS) and Physical Component Summary (PCS) scores. Of note, SF6D is suitable for calculating QALYs and QALDs whereas the MCS and PCS are not (20)**.** SF6D Index Scores at each timepoint were combined with the interval between timepoints to calculate QALDs using an area under the curve approach, assuming a linear trend between timepoints.

Treatment effects with regard to QALDs were estimated on an intention-to-treat basis, with missing values for QALDs (due to missing SF6D scores at any timepoint) replaced using sequential imputation of SF6D scores at each timepoint based on SF6D scores at other timepoints, their relationship to total QALDs and other outcomes, and duration between timepoints. Additional detail regarding imputation of missing values is provided in Appendix S1; Supplementary Table S3.

QALDs reflect changes in SF6D-scores from baseline to final follow-up and adjust for any between-group differences in SF6D scores at baseline. QALDs make no such adjustment for between-group differences in duration to final follow-up and so further adjustment for any such differences is required during analysis. We therefore estimated treatment effects in respect of total QALDs on imputed data, adjusting for residual differences in duration to post-treatment follow-up, baseline SF6D scores, and baseline symptomatology (21).

We also evaluated cost-effectiveness for the Pittsburgh Sleep Quality Index (PSQI; 16) and the FSS [17]. Higher PSQI scores indicate subjectively poorer sleep quality on the PSQI’s 0–21 range (16). To inform interpretation of participant characteristics and estimated treatment effects, the PSQI has a clinical cut-off of > 5 for sleep disorder and a clinically meaningful change of ≥ 3 (16). The FSS measures the impact of fatigue on daily living, with ≥ 4 on a 7-point scale indicating clinically significant fatigue (17). For analysis, we reverse-coded both the PSQI and FSS to allow interpretation of between-group differences as improvements in sleep quality and fatigue severity. For the cost-effectiveness analysis, treatment effects in respect of the PSQI and FSS were otherwise estimated using methods identical to the main effectiveness analysis (15). All analyses regarding incremental effectiveness were conducted using Stata/MP 17.0 for Windows (2021; StataCorp LLC, College Station, TX, USA).

### Incremental cost

Total health system cost was calculated as the sum of intervention delivery costs and health service expenditure. Data sources for each cost component are described in detail in Appendix S1; Supplementary Tables S1, S2, and S4. Treatment effects regarding total health system cost from baseline to final follow-up (overall total cost) were estimated on an intention-to-treat basis, with missing values (due to missing cost components at any timepoint) replaced using sequential imputation of overall total cost based on total costs at each timepoint, their relationship to overall total cost and other outcomes, and duration between timepoints. Additional detail regarding imputation of missing values is provided in Appendix S1: Supplementary Table S3. Treatment effects in respect of overall total cost were estimated on imputed data, adjusting for duration from to post-treatment follow-up and baseline symptomatology (21). All analyses regarding incremental cost were conducted using Stata/MP 17.0 for Windows.

### Productivity gains/losses

Productivity gains/losses were calculated using the human capital approach based on a 1-week activity diary completed at each timepoint. Treatment effects with regard to productivity gains were estimated on an intention-to-treat basis on imputed data, adjusting for covariates. All analyses regarding productivity gains/losses were conducted using Stata/MP 17.0 for Windows.

### Adjustment for differential timing

For the within-trial analysis presented here, all costs and consequences occurred from baseline to final follow-up (average 257 days) and so treatment effects were calculated for undiscounted costs and undiscounted benefits. The study period ran from June 2017 to October 2023 but costs were expressed in 2023/24 A$, using 2023/24 Australian unit costs to calculate the dollar-value of resources (see Appendix S1; Supplementary Table S4).

### Incremental cost-effectiveness

Cost-effectiveness of CBT-SF vs HE was expressed as incremental cost-effectiveness ratios (ICERs) for clinical and economic outcomes. Specifically, we report: (*i*) additional costs (savings) per point difference in PSQI scores at each timepoint, (*ii*) additional costs (savings) per point difference in FSS scores at each timepoint, and (*iii*) additional costs (savings) per QALDs gained to final follow-up. Point estimates for incremental cost-effectiveness were calculated as the average treatment effect on total cost per participant divided by the average treatment effect on the relevant outcome. For comparisons against TAU, we calculated thresholds at which improvements in health service expenditure, productivity, and health outcomes would compensate for the higher incremental cost of delivery relative to TAU.

### Uncertainty

Confidence intervals around ICERs were derived using Fieller’s method based on estimated treatment effects from our main analyses, and standard errors and correlations from bootstrap re-estimation of incremental cost and incremental effectiveness (22). Cost-effectiveness acceptability curves (CEACs) were used to visualize uncertainty associated with the adoption/treatment decision. CEACs were derived using iprogs.do (22) based on estimated treatment effects from our main analyses, and standard errors and correlations data generated from bootstrap re-estimation of treatment effects implemented using bmultiv.do (22) in Stata/MP 17.0 for Windows.

CEACs plot the proportion of the density for which the intervention is cost-effective when the (uncertain) funding threshold is varied over a plausible range. To guide interpretation, we report (*i*) the proportion of the density below the threshold for conservative point estimates of the funding threshold (as described below), and (*ii*) the dollar-value that the (uncertain) funding threshold would have to be in order for 95% of the density to sit below the threshold.

Estimates of funding thresholds for improvements in sleep quality are not currently available. For fatigue severity, results from a recent discrete choice experiment suggest that primary care patients value improvements in “hangover” symptoms of insomnia (feeling groggy, reduced ability to concentrate) at about US$200 per month (23) or about A$390 at 2023 values. For QALDs, 1 recent study (24) estimated the supply-side funding threshold for the Australian health care system at A$28,033 (95% CI: A$20,758–A$37,667) per quality-adjusted life *year* (QALY), which is much lower than alternative estimates (25–27). Recent estimates that recognize both funding constraints and community expectations estimate the cost-effectiveness threshold for Australia at US$49,211 per QALY (95% CI: US$41,884–US$61,634), or A$76,453 per QALY (28). Expressed in terms of quality-adjusted life *days*, interventions with incremental cost-effectiveness ratios up to A$200 can be safely assumed to offer an acceptable balance of costs and benefits. Thresholds for sleep quality and fatigue severity are much more uncertain but A$200 per point improvement on the FSS or PSQI sits within the plausible range of values.

Sensitivity analyses were conducted to evaluate the robustness of study findings to: inclusion/exclusion of productivity costs, and higher/lower unit costs for components of health service utilization (see Appendix S1; Supplementary Table S4).

## Results

Table I summarizes baseline characteristics of participants randomized to treatment and control conditions. Treatment and control groups were comparable at baseline on demographics, injury characteristics and economic outcomes, and proportion enrolled for telehealth delivery, with residual differences on symptomatology and health-related quality of life.

**Table I:** Patient characteristics by treatment condition at baseline

Factor	CBT-SF (*n* = 86)			HE (*n* = 40)			*p*-value
	*n*	Mean (SD), %	Range	*n*	Mean (SD), %	Range	
Demographics							
Age at study entry	86	47.06 (14.62)	20–72	40	49.47 (13.75)	20–70	0.383
Sex (% male)	86	53%	–	40	50%	–	0.715
Years of education	86	13.57 (1.87)	10–18	39	13.46 (2.32)	9–17	0.782
Mode (% telehealth)	86	76%	–	40	78%	–	0.814
Injury characteristics							
Injury type (% TBI)	86	42%	–	40	38%	–	0.643
Time since injury (months)	85	52.74 (56.77)	3.4–252.0	39	63.64 (71.31)	5.1–287.8	0.363
Injury severity (for TBI)	36	Mild (16.7%) Moderate (27.8%) Severe (55.6%)	–	13	Mild (7.7%) Moderate (38.5%) Severe (53.9%)	–	0.638
PTA in days (for TBI)	28	19.75 (25.23)	< 1–109	8	12.00 (12.06)	< 1–29	0.409
GCS score (for TBI)	30	9.33 (4.78)	3–15	11	9.18 (5.02)	3–15	0.930
Stroke mechanism (% ischaemic)	48	69%	–	24	71%	–	0.856
Stroke hemisphere (%)	48	Right (40%) Left (41%) Bilateral (19%)	–	24	Right (54%) Left (21%) Bilateral (25%)	–	0.216
Psychological measures							
Baseline HADS-A	86	8.41 (4.08)	0–20	38	6.55 (3.94)	0–14	0.020
Baseline BPI	41	4.20 (1.62)	1.2–7.5	13	3.12 (1.57)	1.2–7.5	0.039
Economic outcomes							
Baseline HRQoL, SF6D	86	0.63 (0.11)	0.43–0.96	38	0.66 (0.10)	0.52–0.87	0.104
% Productive time	82	70.3 (19.4)	24–100	36	68.7 (18.2)	35–96	0.677
Any medication	86	73.3%	–	40	77.5%	–	0.614
Medication for sleep	86	9.3%	–	40	12.5%	–	0.586
Medication for alertness	86	3.5%	–	40	2.5%	–	0.771
Medication for mood	86	26.7%	–	40	27.5%	–	0.930
Medication for pain	86	9.3%	–	40	15.0%	–	0.348

GCS: Glasgow Coma Scale; CBT-SF: cognitive behaviour therapy for sleep and fatigue; HE: health education; PTA: post-traumatic amnesia; HADS-A: Hospital Anxiety and Depression Scale – Anxiety subscale; BPI: Brief Pain Inventory; SD: standard deviation; TBI: traumatic brain injury; SF6D: Short-Form Six Dimension; HRQoL: health-related quality of life.

### Incremental effectiveness

Table II reports treatment effects with regard to our economic outcomes (health-related quality of life at each time-point as measured by the SF6D and SF6D-based QALDs to final follow-up) and summarizes results from the main effectiveness analysis in respect of clinical outcomes (reverse-coded PSQI and FSS) that underpin the economic evaluations reported here. For the primary clinical outcome of sleep quality, predicted differences between treatment and control groups on the PSQI reached statistical significance at post-treatment follow-up (mean difference: 1.78, 95% CI: 0.16, 3.41) and remained in favour of the treatment group at 2 months post-treatment (mean difference: 0.46, 95% CI: –1.17, 2.10) but not at final follow-up (mean difference: –0.18, 95% CI: –1.84, 1.48). For fatigue severity, predicted differences between treatment and control groups on the FSS were in favour of the treatment group at all timepoints and reached statistical significance at 2 months post-treatment (mean difference: 0.58, 95% CI: 0.09, 1.08).

**Table II:** Effect of the intervention on clinical outcomes and quality of life

Outcome	Mean, predicted^f^ (SE)		Increment, predicted^g^ (95% CI)
	CBT-SF	HE	
Clinical outcomes			
PSQI^a^, Post-Tx	6.39 (0.45)	8.17 (0.69)	**1.78 (0.16, 3.41)**
PSQI, M2	6.88 (0.46)	7.34 (0.69)	0.46 (–1.17, 2.10)
PSQI, M4	6.67 (0.46)	6.49 (0.70)	–0.18 (–1.84, 1.48)
FSS^b^, Post-Tx	4.78 (0.14)	5.26 (0.21)	0.48 (–0.01, 0.97)
FSS, M2	4.68 (0.14)	5.27 (0.21)	**0.58 (0.09, 1.08)**
FSS, M4	4.74 (0.14)	4.99 (0.21)	0.25 (–0.26, 0.75)
Quality of life, SF6D index scores			
SF6D^c^, Post-Tx	0.68 (0.01)	0.65 (0.01)	**0.03 (–0.00, 0.06)**
SF6D, M2	0.68 (0.01)	0.66 (0.01)	0.02 (–0.01, 0.05)
SF6D, M4	0.69 (0.01)	0.71 (0.01)	–0.02 (–0.05, 0.02)
Quality of life, QALDs^d^			
Total QALDs, base case^e^	178.70 (2.78)	175.33 (3.67)	3.37 (–4.18, 10.92)

Post-Tx: post-treatment follow-up; M2: 2 months post-treatment; M4: 4 months post-treatment.

a Pittsburgh Sleep Quality Index (PSQI) scores at relevant timepoint.

b Fatigue Severity Scale (FSS) scores at relevant timepoint.

c SF36-based SF6D scores at relevant timepoint.

d Marginal means and between-group difference in respect of total QALDs estimated via linear regression on imputed data *adjusting for* duration from baseline to post-treatment follow-up, baseline SF6D scores, and baseline symptomatology as measured by the Hospital Anxiety and Depression Scale. Estimates derived from *mimrgns r.group* and *mimrgns i.group*, *predict(xb)* after *mi estimate: regress outcome i.group covariates.*

e Quality-Adjusted Life Days (QALDs) calculated based on SF6D index scores at each timepoint using an area under the curve approach, assuming a linear trend between timepoints for each patient.

f Mean, predicted = marginal means by group for each timepoint based on main and interaction effect for treatment group and time; adjusted for baseline symptomatology in the case of clinical outcomes; and for baseline symptomatology and baseline quality of life in the case of SF6D; and for baseline symptomatology, baseline quality of life, and duration from baseline to post-treatment follow-up in the case of QALDs. Treatment effects by timepoint for PSQI and FSS in the main effectiveness analysis. Estimates derived from *margins i.group##i.timepoint*, *predict(xb)* after *mixed outcome i.group##i.timepoint covariates || participant*:, *reml*. For clinical outcomes, identical estimates of marginal means by group are reported in Figs 2 and 3 of the main effectiveness paper (6).

g Increment, predicted = marginal effect of treatment group for each timepoint based on main and interaction effect for treatment group and time; adjusted for baseline symptomatology in the case of clinical outcomes; and for baseline symptomatology and baseline quality of life in the case of SF6D; and for baseline symptomatology, baseline quality of life, and duration from baseline to post-treatment follow-up in the case of QALDs. Estimates derived from margins r.group, over(timepoint) after mixed outcome i.group##i.timepoint covariates || participant:, reml. Results in bold significant at 0.01, 0.05 or 0.001 level.

Improvements in sleep quality and fatigue severity contribute to broader improvements in health-related quality of life as measured by the SF6D at post-treatment (mean difference: 0.03, 95% CI: –0.00, 0.06) and at 2 months post-treatment (mean difference: 0.02, 95% CI: –0.01, 0.05), reaching statistical significance at the 10% level for health-related quality of life at post-treatment (*p* = 0.086). Combining SF6D scores at each timepoint with duration gives us a summary of the incremental health effects of CBT-SF over HE. The predicted increment of 3.37 quality-adjusted life days per participant is estimated imprecisely (95% CI: –4.18, 10.92) but well within the bounds of clinical significance and may serve to offset (in whole or in part) the direct cost of the intervention.

### Incremental cost

Appendix S1; Tables S1.1 and S2.1) summarize the total direct and indirect costs of delivering CBT-SF (mean: A$2,767, SD: A$558) and HE (mean: A$2,435, SD: A$654). HE is an active intervention and so the incremental direct and indirect cost of delivering CBT-SF over HE (mean difference: A$333, 95% CI: A$109, A$556) is unlikely to provide a realistic estimate of the additional cost of CBT-SF over TAU in wider implementation. For both CBT-SF and HE, the total direct and indirect cost of delivery includes: per participant labour and time costs for delivery and receipt of CBT-SF and HE sessions, per participant overhead costs associated with receipt of telemedicine and in-person sessions, per participant travel costs for receipt of in-person sessions, and per participant fixed cost of training, supervision, and materials necessary for delivery of the CBT-SF and HE interventions. Each of these cost components is additional to TAU and our best estimate of the *incremental* direct and indirect cost of delivering CBT-SF *over TAU* is just the *total* direct and indirect cost of delivering CBT-SF (mean: A$2,767, 95% CI: A$2,648, A$2,887).

Table III summarizes our base-case, pessimistic, and optimistic comparisons of CBT-SF and HE groups with regard to health service expenditure (excluding direct and indirect costs of delivery) and total health system cost (including direct and indirect costs of delivery). Base-case estimates reflect our best available estimate of unit costs and utilization for components of health service utilization. Pessimistic (optimistic) estimates reflect uncertainty regarding unit costs and/or utilization, and have the effect of delating (inflating) observed differences in health service expenditure between treatment and control groups.

**Table III:** Effect of the intervention on health service expenditure, total health system cost, and productivity gains/losses

Outcome	Mean, predicted^e^ (SE)		Increment, predicted (95% CI)
	CBT-SF	HE	
Health service expenditure^a^			
Base case	$3,997 (881)	$4,774 (1,329)	–$777 (–4,232, 2,678)
Optimistic	$6,337 (1,688)	$7,380 (2,742)	–$1043 (–7,632, 5,547)
Pessimistic	$5,082 (971)	$5,430 (1,639)	–$349 (–4,152, 3,455)
Total health system cost^b^			
Base case	$6,947 (878)	$7,428 (1,324)	–$481 (–3,921, 2,960)
Optimistic	$9,059 (1,663)	$9,932 (2,879)	–$873 (–7,781, 6,035)
Pessimistic	$8,031 (968)	$8,084 (1,633)	–$54 (–3,844, 3,736)
Productivity gains/losses^c^			
Value of productive time^d^	$48,476 (1,009)	$47,759 (1,445)	$717 (–2,775, 4,208)

a Per patient health service expenditure to final follow-up, calculated as the price-weighted sum of patient-reported use of medications, medical and allied health services, and hospitalizations for intervals between timepoints.

b Sum of per patient intervention costs for the CBT-SF or HE interventions and per patient health service expenditure to final follow-up.

c Marginal means and between-group difference calculated as for health service expenditure and total health system cost but estimated adjusting for additional covariates (baseline productive time and baseline pain).

d Per patient productivity gains/losses to final follow-up calculated using an area-under-the-curve approach, assuming a linear trend between timepoints for each patient’s percentage time spent in productive activity, applying this percentage to a standard 40-hour working week and multiplying by the average all-industries hourly wage rate.

e Marginal means and between-group difference in respect of cost components estimated via linear regression on imputed data *adjusting for* duration from baseline to post-treatment follow-up and baseline symptomatology as measured by the Hospital Anxiety and Depression Scale. Estimates derived from *mimrgns r.group* and *mimrgns i.group*, *predict(xb)* after *mi estimate: regress outcome i.group covariates*.

In the base case, total health service expenditure per participant in the CBT-SF group (mean: A$3,997, SE: A$881) was lower than in the HE group (mean: A$4,774, SE: A$1,329); though this difference did not approach statistical significance (mean difference: –A$777, 95% CI: –A$4,232, A$2,678). Rolling in the direct and indirect costs of delivery (reported above) to obtain total health system cost in CBT-SF (mean: A$6,948, SE: 878) and HE groups (mean: A$7,428, SE: 1,324), reduces the net savings from receipt of the intervention (mean difference: –A$481, 95% CI: –3,921, 2,960). Results from the optimistic and pessimistic sensitivity analyses were qualitatively consistent with base-case estimates (see Table III and Appendix S1; Supplementary Table S5).

Of note (and as above), the incremental cost of delivering CBT-SF is *higher* relative to TAU than when compared with HE. Incremental health service expenditure under CBT-SF may, however, be *lower* relative to TAU – yielding *larger cost-savings* – than relative to HE because HE is an active intervention that may reduce health expenditure associated with sleep disturbance and fatigue (29). Note that both CBT-SF and HE groups showed significant reductions in sleep and fatigue symptoms by 4 months post-treatment in the main effectiveness analysis (15).

These 2 effects on cost of delivery and on health service expenditure operate in *opposite directions* and both would need to be captured when calculating incremental health system cost relative to TAU. Without further information regarding impacts on health service expenditure, we calculate total health system cost under the assumption that HE has no impact on health expenditure associated with sleep disturbance and fatigue. Under this assumption, total health system cost under CBT-SF (mean: A$6,944, SE: A$879) group would be somewhat higher than under TAU (mean: A$4,765, SE: A$1,326) and wider roll-out of CBT-SF would impose a net cost on the health system (mean difference: A$2,178, 95% CI: –1,268, 5,624). If we were instead to assume that health service expenditure is at least A$2,178 higher under TAU than under HE, then wider-roll out of CBT-SF would remain cost-saving relative to TAU.

### Productivity gains/losses

When time spent in productive activity to final follow-up was valued in dollar terms, productivity gains in the CBT-SF group (mean: A$48,476, SE: A$1,009) were marginally larger than in the HE group (mean: A$47,759, SE: A$1,445); though this difference did not approach statistical significance (mean difference: A$717, 95% CI: –A$2,775, A$4,208). Productivity gains are second-order considerations for many fundholders, particularly when estimated with this degree of uncertainty (30). We therefore limit our reporting of incremental cost-effectiveness to the health system perspective.

### Incremental cost-effectiveness

Results from the cost-effectiveness analyses under base-case assumptions are reported in Table IV. Results from optimistic and pessimistic sensitivity analyses are broadly consistent with base-case analyses and reported in Appendix S1. When taken together, significant improvements in sleep quality and health-related quality of life at post-treatment follow-up and significant improvements in fatigue severity at 2 months post-treatment suggest that CBT-SF for sleep disturbance and fatigue after ABI may be cost-effective if it comes at an acceptable cost. At the mean, CBT-SF dominates (less costly and more effective) HE but the proportion of the density (joint distribution of cost and outcomes) considered cost-effectiveness varies depending on the cost-effectiveness threshold.

**Table IV:** Incremental cost-effectiveness, base case

Variable	∆C	∆E	∆C/∆E	% acceptable at $200 threshold^a^	Threshold for 95% confidence^b^
Clinical outcome^c^					
Cost per PSQI^d^, Post-Tx	–$481 (–3,921, 2,960)	**1.78 (0.16, 3.41)**	Tx dominates	73%	$1,551
Cost per PSQI, M2	–$481 (–3,921, 2,960)	0.46 (–1.17, 2.10)	Tx dominates	66%	Undefined^e^
Cost per FSS^f^, Post-Tx	–$481 (–3,921, 2,960)	0.48 (–0.01, 0.97)	Tx dominates	66%	$5,447
Cost per FSS, M2	–$481 (–3,921, 2,960)	**0.58 (0.09, 1.08)**	Tx dominates	67%	$4,662
Quality of life, QALDs					
Cost per QALD^g^	–$481 (–3,921, 2,960)	3.37 (–4.18, 10.92)	Tx dominates	76%	Undefined^e^

Post-Tx: post-treatment follow-up; M2: 2 months post-treatment; M4: 4 months post-treatment. Results in bold significant at 0.01, 0.05 or 0.001 level.

a Percentage of the joint density of costs and outcomes below the $200 funding threshold where the density is derived via Fieller’s method using iprogs.do (22).

b Dollar value that the (uncertain) funding threshold would have to be in order for 95% of the density to sit below the threshold.

c Calculated per point improvement (–DE) to reflect the fact that relative *reduction* in PSQI and FSS reflects an improvement.

d Cost per point improvement in PSQI scores at the relevant timepoint calculated using base-case estimates of treatment effects for total health system cost to final follow-up and PSQI at the relevant timepoint.

e Widest definable confidence interval < 95%.

f Cost per point improvement in FSS scores at the relevant timepoint calculated using base-case estimates of treatment effects for total health system cost to final follow-up and FSS at the relevant timepoint.

g Cost per QALD calculated using base-case estimates of treatment effects for total health system cost and QALDs to final follow-up.

**Fig. 1:**
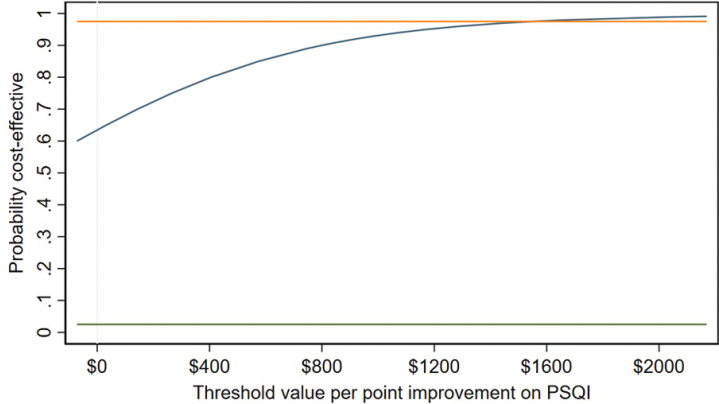
Cost-effectiveness acceptability curve for cost per point improvement in Pittsburgh Sleep Quality Index (PSQI) scores post-treatment.

At a cost-effectiveness threshold of A$200 for each of our clinical and economic outcomes, the proportion of the density deemed cost-effective ranges from 66% to 76% under base-case assumptions. For cost per point improvement on the PSQI at post-treatment follow-up, 73% of the density is cost-effective at the A$200 threshold but this increases to 95% of the density at a threshold of A$1,551 per point improvement. Fig. 1 plots the cost-effectiveness acceptability curve (CEAC), illustrating the gradient in probability of cost-effectiveness as the cost-effectiveness threshold varies from A$0 to A$2,000. While A$2,000 per point improvement on the PSQI is unlikely to represent good value for money, the CEAC reaches 80% of the density at a threshold of just over A$400.

**Fig. 2:**
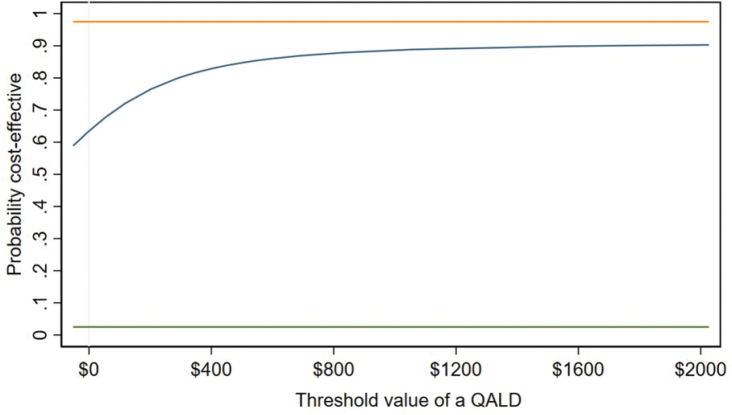
Cost-effectiveness acceptability curve for cost per Quality-Adjusted Life Day (QALD) to final follow-up.

For cost per QALD to final follow-up, 76% of the density is cost-effective at the A$200 threshold. Inspection of the CEAC in Fig. 2 demonstrates that further increases in confidence are forthcoming at higher thresholds but there is no threshold at which we can be 95% confident that CBT-SF is more cost-effective than HE.

## DISCUSSION

### Principal findings

The addition of CBT-SF to usual care for sleep disturbance and fatigue following ABI carries 3 potential benefits. First, improvements in sleep quality, fatigue, and associated symptomatology may carry value to people with ABI and society as a final outcome or for their impact on quality of life. Second, improvements in sleep quality, fatigue, and associated symptomatology may reduce the need for and use of health services to manage these conditions, resulting in cost-savings to people with ABI and the health system. Third, improvements in sleep quality, fatigue, and associated symptomatology may translate into improvements in capacity to participate in household, economic, or social production. The present study quantified the trade-off between the incremental cost of delivering CBT-SF and its potential benefits from health system and societal perspectives.

Results suggests that CBT-SF is likely to be cost-effective for reducing sleep disturbance and fatigue and improving health-related quality of life after TBI and stroke. Of note, this result is based on comparison against HE rather than TAU. At the mean, reductions in health service expenditure are sufficient to offset the incremental cost of CBT-SF over HE such that quantitatively larger improvements in sleep quality (post-treatment), fatigue (at 2 months post-treatment), and health-related quality of life (post-treatment) under CBT-SF are likely available at an acceptable cost. When uncertainty in respect of treatment effects is characterized relative to the uncertain cost-effectiveness threshold, the probability that CBT-SF is cost-effective in comparison with HE ranges from 66% to 77% in the base case. Even in our most pessimistic sensitivity analysis, CBT-SF remains more likely to be cost-effective than our HE comparator.

The comparison against HE is unlikely to provide a realistic estimate of the additional cost of CBT-SF over TAU in wider implementation. Both CBT-SF and HE are active interventions such that health outcomes may be better, productivity gains larger, and health service expenditure lower under HE than under TAU (29). Results from the present study suggest that HE can also be expected to be more costly to deliver than TAU, with a net impact on health outcomes, productivity gains, and health service expenditure equivalent to A$2,178 in savings or benefits required to compensate for the higher cost of delivery. The magnitude of treatment effects relative to HE reported in the present study suggests that cost-effectiveness relative to TAU is well within reach.

Productivity gains in the CBT-SF group were marginally larger than in the HE group but there was significant uncertainty regarding the magnitude of this effect. Findings regarding cost-effectiveness set these gains aside but are sufficient to suggest that evaluation from a health system perspective may offer a conservative estimate of net benefits to society.

### Study limitations

First, there are limits to the generalizability of our findings. Our estimates of incremental cost and incremental cost-effectiveness partly reflect protocol-driven costs due to delivery of the intervention and control conditions within the context of a clinical trial. In routine practice, there may be scope to deliver less intensive care without compromising patient outcomes. Other limits on generalizability follow from the choice to conduct our evaluation in a particular setting and from a particular perspective. For example, trial recruitment and follow-up spanned the period June 2017 to October 2023, encompassing significant periods of COVID-related travel restrictions during 2020/2021. While we adjust for temporal variation in prices, COVID-related travel restrictions likely contributed to the higher than expected proportion of telehealth participants in our study sample. More generally, our estimates of cost and cost-effectiveness reflect the cost of goods and services in Australia. In other settings, the cost of delivering the CBT-SF and HE conditions and of broader health service utilization may be somewhat lower (or higher) than in Australia and this may alter the relative cost and cost-effectiveness of our intervention and control conditions.

Second, comparison against an active control rather than TAU complicates the task of characterizing costs and benefits of implementing CBT-SF for sleep disturbance and fatigue in routine practice. While previous trials (4, 5) showed CBT-SF to be superior to TAU and the present trial provides indirect information regarding incremental costs relative to TAU, further research may be required to estimate cost-effectiveness relative to TAU precisely.

Finally, our results reflect average costs and effectiveness across TBI and stroke cohorts. Results from the main effectiveness analysis suggest that the balance of costs and benefits may be more favourable in TBI than this average would suggest (15).

### Conclusion

CBT-SF is likely to be cost-effective relative to HE for reducing sleep disturbance and fatigue and improving health-related quality of life after TBI and stroke. Adding CBT-SF to usual care would also be cost-effective under realistic assumptions regarding the relative impact of HE and TAU on health outcomes, productivity gains, and health service expenditure. Further research should precisely characterize the cost-effectiveness of CBT-SF vs TAU in clinically relevant sub-groups.

## Supplementary Material

COST-EFFECTIVENESS OF COGNITIVE BEHAVIOURAL THERAPY VERSUS HEALTH EDUCATION FOR SLEEP DISTURBANCE AND FATIGUE FOLLOWING STROKE AND TRAUMATIC BRAIN INJURY
