# Supplementary material for: COST-EFFECTIVENESS OF COGNITIVE BEHAVIOURAL THERAPY VERSUS HEALTH EDUCATION FOR SLEEP DISTURBANCE AND FATIGUE FOLLOWING STROKE AND TRAUMATIC BRAIN INJURY
Source: J Rehabil Med. 2025 Apr 24;57:42770. doi: 10.2340/jrm.v57.42770 (PMC12041795; doi:10.2340/jrm.v57.42770)
Supplement: COST-EFFECTIVENESS OF COGNITIVE BEHAVIOURAL THERAPY VERSUS HEALTH EDUCATION FOR SLEEP DISTURBANCE AND FATIGUE FOLLOWING STROKE AND TRAUMATIC BRAIN INJURY [file JRM-57-42770-s1.pdf]

Supplementary material has been published as submitted. It has not been copyedited, or typeset by Journal of Rehabilitation Medicine

Appendix S1

Supplemental Material S1

Resource-based costing for delivery of the intervention

Community dwelling adults with stroke or traumatic brain injury were randomized 2:1 to intervention (*n*=86) or control (*n*=40). Participants randomized to the intervention group received 8-weeks of manualized Cognitive Behavioral Therapy treatment for Sleep and Fatigue (CBT-SF) that included weekly, one-on-one sessions of CBT-SF with a neuropsychologist plus usual care. The eight weekly sessions were structured around seven modules designed to address maladaptive beliefs and behaviors perpetuating sleep and fatigue symptoms with session delivery and therapeutic goals tailored to each participant's presentation and priorities. CBT-SF participants were provided with written materials to support and reinforce information and techniques discussed in session. Individualized homework tasks were assigned at each CBT-SF session and reviewed weekly by the treating therapist. Therapists charged with delivery of the CBT-SF intervention (and of the HE intervention described in Supplemental Materials S2) were qualified neuropsychologists with higher qualifications and received study-specific training and supervision regarding delivery of the manualized intervention. CBT-SF sessions were delivered face-to-face (24%) or via videoconferencing (76%) according to participant preference, with randomization *after* baseline and *after* selecting mode of delivery, using separate randomization schedules for each mode.

The CBT-SF intervention was developed and manualized to improve fidelity / replicability and facilitate implementation. While this entailed significant resource use, the associated costs are now 'sunk' and decision-makers must now decide whether or not to adopt the manualised intervention as recommended practice. For this reason, we evaluate the cost-effectiveness of intervention delivery (*ex post* of intervention development) and exclude intervention development costs from the cost analysis.

Outcomes were assessed in both treatment and control groups at baseline, post-treatment, 2-months post-treatment and 4-months post-treatment, and researchers conducting follow-ups were blinded to treatment allocation. Costs of outcome assessment were therefore invariant between treatment and control groups and so we set costs associated with outcome assessment aside as research costs. Likewise, treatment fidelity was evaluated via audio recordings rather than in-person observation and so researcher time in reviewing and scoring audio recordings for fidelity was unlikely to directly influence quality of care. We therefore exclude costs associated with fidelity monitoring from our analysis.

Total cost per patient from the health system perspective was calculated as the sum of (i) per participant intervention costs for the CBT-SF intervention (based on participant-level administrative data regarding adherence to the treatment protocol and average therapist hours in intervention-specific training), and (ii) the per participant cost of medical services and rehabilitation therapy to final follow-up (based on

participant-level self-report data regarding use of medications, medical and allied health services and hospitalisations at each timepoint). Per participant productivity costs from baseline to final follow-up were evaluated (as described below) to inform evaluation from the societal perspective. Table S1 summarises the resource requirements for *delivery* of the CBT-SF intervention as trialled. Table S3 in Supplementary Material S3 provides unit costs for line items specified in Table S1, as well as further explanation regarding data sources and assumptions for calculation of the per participant cost of medical services and rehabilitation therapy.

Participant and therapist time in delivery/receipt of treatment are inputs in the production of health services and so we include these in estimates of total health system cost, though arguments could be made for their exclusion (1). Our estimate of total health system costs also includes minor travel costs and overheads associated with receipt of the CBT-SF and HE conditions. While quantitatively small, such costs would typically be reserved for inclusion under a patient or societal perspective such that our estimate of total health system cost is only differentiated from total societal cost by the exclusion of productivity gains/losses.

Productivity gains/losses were calculated using the human capital approach based on a one-week activity diary completed at each timepoint. Diary entries for physical and mental activity, rest and sleep were converted into a percentage time spent in productive activity. Productivity gains/losses expressed in units of time (hours) were then calculated for the full study period using an area-under-the-curve approach, assuming a linear trend between time-points for each participant's percentage time spent in productive activity and applying this percentage to a standard 40 hour working week. Productivity gains/losses expressed in dollar terms were calculated by multiplying the number of hours in productive activity by the average all industries hourly wage rate for May 2023 (2).

Of note, we apply the same dollar-value to hours in paid employment and hours in other productive activity where the average hourly wage rate effectively provides a shadow price for hours in unpaid productive activity. This is consistent with methodological guidelines that recommend the inclusion of, "…not only productivity losses related to absenteeism from and reduced productivity at paid work, but also those related to unpaid work" (3) and broadly accepted definitions of productivity costs as "costs associated with production loss and replacement costs due to illness, disability and death of productive persons, *both paid and unpaid* " (4). Valuation of both paid and unpaid contributions obviates the need to censor productivity gains/losses following a friction period or discount productivity gains/losses due to short-term adjustments by employers (5).

Supplemental Table S1: Per participant cost of delivery for CBT-SF, 2023/24 AUD

Input	Number (A)	Unit cost (B)	Total cost (A x B)	Per participant (pt) cost (A x B / n=86)
**Therapist training & supervision**				
Familiarisation and training in manualised intervention, therapist time	18.5 hrs / therapist for 7 CBT-SF therapists = 129.5 hrs	$57.17 / hour	$7,403.52	$86.09 / pt
One-on-one supervision, therapist time	40 hrs^ / therapist for 7 therapists = 280 hrs	$57.17 / hour	$16,007.60	$186.13 / pt
One-on-one supervision, supervisor time	40 hrs total^	$69.27 / hour	$2,770.80	$32.22 / pt
Group supervision sessions, therapist time	30 hrs / therapist for 7 therapists = 210 hrs	$57.17 / hour	$12,005.70	$139.60 / pt
Group supervision sessions, supervisor time	84 sessions* with 2 supervisors per session = 168 hrs	$69.27 / hour	$11,637.36	$135.32 / pt
		Sub-total	$49,824.98	$579.36 / pt
**Delivery of the CBT-SF Intervention**				
CBT-SF sessions, participant time	Average 7.4 sessions / pt, (sd: 1.9; min: 0, max: 8)	$44.00 / hour	$28,160.00	Average $327.44 / pt
CBT-SF sessions, participant travel time for face-to-face sessions	Average 1.7 sessions / pt (sd: 3.3; min: 0, max: 8) delivered face-to-face, 45 mins travel time per session	$44.00 / hour	$4,917.00	Average $57.17 / pt
CBT-SF sessions, participant overhead costs for face-to-face sessions	Average 1.7 sessions / pt (sd: 3.3; min: 0, max: 8) delivered face-to-face	$5.30 / session	$789.70	Average $9.18 / pt

Input	Number (A)	Unit cost (B)	Total cost (A x B)	Per participant (pt) cost (A x B / n=86)
CBT-SF sessions, participant overhead costs for telehealth sessions	Average 5.7 sessions / pt (sd: 3.6; min: 0, max: 8) delivered via telehealth	$1.90 / session	$932.90	Average $10.85 / pt
CBT-SF sessions, therapist time	Average 7.4 sessions / pt (sd: 1.9; min: 0, max: 8) with 1 hour delivery time & 15 mins prep per session	$190.03 / hour	$152,023.99	Average $1,767.72 / pt
Materials, printed hand-outs and homework	Up to 63 pages per pt of colour printed A4 handouts and homework sheets	$0.25 / side	$1,354.50	$15.75 / pt
		Sub-total	$188,178.15	$2,188.12 / pt
		Grand Total	**$238,003.11**	**$2,767.48 / pt**

^Total of 60 hrs one-on-one supervision time across both groups apportioned in 2:1 ratio across treatment and control groups, as per randomisation schedule.

*Group supervision sessions were conducted with 2-3 therapists per session and 2 supervisors, requiring 84 sessions to provide 30 hours of group supervision per therapist.

Supplemental Material S2

Cost analysis for delivery of the comparator intervention

Participants randomized to the control group received an 8-week Health Education (HE) intervention that included weekly one-on-one sessions of HE with a neuropsychologist plus usual care. HE sessions were delivered face-to-face (22%) or via videoconferencing (78%) according to participant preference and presented as an active intervention. HE participants were not assigned individualized homework tasks but were given written handouts and take-home worksheets to complete and the HE intervention was otherwise structured identically to CBT-SF.

Delivery of an active intervention (HE) to the control group may compensate for non-specific therapy factors and any placebo effect in the intervention group. As a consequence, the control condition no longer reflects usual care in routine clinical practice. For this reason, supplementary analyses were conducted in which costs of HE were excluded from the cost-analysis but estimates of incremental effectiveness and cost-effectiveness were otherwise based on CBT-SF versus HE treatment effects. This supplementary analysis was designed to provide a more realistic estimate of incremental cost in wider implementation of CBT-SF but may *underestimate* incremental effectiveness and incremental cost-effectiveness relative to usual care.

The HE intervention was developed and manualized ostensibly for research purposes. While this entailed significant resource use, the associated costs are now ‘sunk’ and decision-makers must now decide whether or not to adopt the evaluated intervention as recommended practice. For this reason, we evaluate the cost-effectiveness of intervention delivery (*ex post* of intervention development and *ex post* of developing and manualising the active control condition) and exclude development costs from the cost analysis.

Outcomes were assessed in both treatment and control groups at baseline, post-treatment, 2-months post-treatment and 4-months post-treatment, and researchers conducting follow-ups were blinded to treatment allocation. Costs of outcome assessment were therefore invariant between treatment and control groups and so we set costs associated with outcome assessment aside as research costs. Likewise, treatment fidelity was evaluated via audio recordings rather than in-person observation and so researcher time in reviewing and scoring audio recordings for fidelity was unlikely to directly influence quality of care. We therefore exclude costs associated with fidelity monitoring from our analysis.

Total cost per patient from the health system perspective was calculated as the sum of (i) per participant intervention costs for the HE intervention (based on participant-level administrative data regarding adherence to the treatment protocol and average therapist hours in intervention-specific training), and (ii) the per participant cost of medical services and rehabilitation therapy to final follow-up (based on participant-level self-report data regarding use of medications, medical and allied health services and hospitalisations at each timepoint).

Total cost per patient from the health system perspective was calculated as the sum of (i) per participant intervention costs for the HE intervention (based on participant-level administrative data regarding adherence to the treatment protocol and average therapist hours in intervention-specific

training), and (ii) the per participant cost of medical services and rehabilitation therapy to final follow-up (based on participant-level self-report data regarding use of medications, medical and allied health services and hospitalisations at each timepoint). Per participant productivity costs from baseline to final follow-up were evaluated (as described below to inform evaluation from the societal perspective). Table S2 summarises the resource requirements for delivery of the HE intervention as trialled. Table S3 in Supplementary Material S3 provides unit costs for line items specified in Table S2, as well as further explanation regarding data sources and assumptions for calculation of the per participant cost of medical services and rehabilitation therapy**.**

Participant and therapist time in delivery/receipt of treatment are inputs in the production of health services and so we include these in estimates of total health system cost, though arguments could be made for their exclusion (1). Our estimate of total health system costs also includes minor travel costs and overheads associated with receipt of the CBT-SF and HE conditions. While quantitatively small, such costs would typically be reserved for inclusion under a patient or societal perspective such that our estimate of total health system cost is only differentiated from total societal cost by the exclusion of productivity gains/losses.

Productivity gains/losses were calculated using the human capital approach based on a one-week activity diary completed at each timepoint. Diary entries for physical and mental activity, rest and sleep were converted into a percentage time spent in productive activity. Productivity gains/losses expressed in units of time (hours) were then calculated for the full study period using an area-under-the-curve approach, assuming a linear trend between time-points for each participant's percentage time spent in productive activity and applying this percentage to a standard 40 hour working week. Productivity gains/losses expressed in dollar terms were calculated by multiplying the number of hours in productive activity by the average all industries hourly wage rate for May 2023 (2).

Of note, we apply the same dollar-value to hours in paid employment and hours in other productive activity where the average hourly wage rate effectively provides a shadow price for hours in unpaid productive activity. This is consistent with methodological guidelines that recommend the inclusion of, "…not only productivity losses related to absenteeism from and reduced productivity at paid work, but also those related to unpaid work" (3) and broadly accepted definitions of productivity costs as "costs associated with production loss and replacement costs due to illness, disability and death of productive persons, *both paid and unpaid* " (4). Valuation of both paid and unpaid contributions obviates the need to censor productivity gains/losses following a friction period or discount productivity gains/losses due to short-term adjustments by employers (5).

Supplemental Table S2: Per participant cost of delivery for HE, 2023/24 AUD

Input	Number (A)	Unit cost (B)	Total cost (A x B)	Per participant (pt) cost (A x B / n=40)
**Therapist training & supervision**				
Familiarisation and training with HE intervention, therapist time	9 hrs / therapist for 7 HE therapists = 63 hrs	$57.17 / hour	$3,601.71	$90.04 / pt
One-on-one supervision, therapist time	20 hrs^ / therapist for 7 therapists = 140 hrs	$57.17 / hour	$8,003.80	$200.10 / pt
One-on-one supervision, supervisor time	20 hrs total^	$69.27 / hour	$1,385.40	$34.64 / pt
		Sub-total	$12,990.91	$324.77 / pt
**Delivery of the CBT-SF Intervention**				
HE sessions, participant session time	Average 7.2 sessions / pt (sd: 2.2; min: 0, max: 8)	$44.00 / hour	$12,716.00	Average $317.90 / pt
HE sessions, participant travel time for face-to-face sessions	Average 1.5 sessions / pt (sd: 3.1; min: 0, max: 8) delivered face-to-face, 45 mins travel time per session	$44.00 / hour	$2,013.00	Average $50.33 / pt
HE sessions, participant overhead costs for face-to-face sessions	Average 1.5 sessions / pt (sd: 3.1; min: 0, max: 8) delivered face-to-face,	$5.30 / session	$323.30	Average $8.08 / pt
HE sessions, participant overhead costs for telehealth sessions	Average 5.7 sessions / pt (sd: 3.6; min: 0, max: 8) delivered via telehealth	$1.90 / session	$433.20	Average $10.83 / pt
HE sessions, therapist time	Average 7.2 sessions / pt	$190.03 / hour	$68,648.34	Average $1,716.21 / pt

Input	Number (A)	Unit cost (B)	Total cost (A x B)	Per participant (pt) cost (A x B / n=40)
	(sd: 2.2; min: 0, max: 8) with 1 hour delivery time & 15 mins prep per session			
Materials, printed hand-outs	26 pages per pt of colour printed A4 handouts	$0.25 / side	$260.00	$6.50 / pt
		Sub-total (base-case)	$84,393.84	$2,109.85 / pt
		Grand Total (base-case)	$97,384.75	$2,434.62 / pt

^Total of 60 hrs one-on-one supervision time across both groups apportioned in 2:1 ratio across treatment and control groups, as per randomisation schedule.

Supplemental Material S3

Imputation of missing values

Table S3 summarises the level of missing data for outcomes at each timepoint. In line with the main effectiveness analysis, missing values on clinical outcomes (PSQI and FSS) were addressed using linear mixed effect models (LMMs). LMMs rely on pair-wise deletion and repeated measures to handle missing data, wherein a patient-specific random effect accounts for the dependence between repeated observations for the relevant outcome (6). LMMs offer a simple alternative to handling missing data *without imputation* under Missing At Random (MAR) assumptions but are *only* an option when we can make use of the panel structure of the data.

For QALDs, costs and productivity gains, we were faced with a choice between using actual or planned duration between time-points to calculate totals over the study period. In the present study, the actual timing of follow-up occasionally varied from planned time-points and – because both costs and QALDs have a time-dimension – we use actual duration between timepoints to calculate totals. This meant that we had to address missing data for duration between timepoints as well as for the outcome of interest at each of the relevant timepoints. While it is possible to calculate QALDs from timepoint to timepoint to obtain repeated measures data for use in LMMs and then calculate total QALDs to trial end by summing over treatment effects for each interval, this approach complicates bootstrap of the joint distribution of costs and effects. It requires summation over marginal effects for each interval from the LMM model after each iteration of the bootstrap routine. For this reason and because multiple imputation with chained equations (MICE) performs relatively well when data is Missing Not At Random (MNAR) rather than MAR (7), we calculated total QALDs and total cost and total productivity gains to trial end for completers and then directly imputed missing values for these totals using multiple imputation with chained equations as described in the main manuscript and in notes to Table S3b.

For each outcome of interest, we specified a parsimonious set of predictors in the multiple imputation routine. Table S3b demonstrates robustness of our findings to inclusion of a larger set of predictors in the MI routine.

Table S3a: Missing values for outcomes and covariates included in treatment effect regressions*

	CBT-SF (n=86)		HE (n=40)	
	Missing (%)	Non-missing (%)	Missing (%)	Non-missing (%)
Duration, Baseline to Post-Tx	9 (10%)	77 (90%)	7 (17.5%)	33 (82.5%)
SF6D, Baseline	0 (0%)	86 (100%)	2 (5%)	38 (95%)
SF6D, Post-Tx	9 (10%)	77 (90%)	7 (17.5%)	33 (82.5%)
SF6D, M2	15 (17%)	71 (83%)	9 (22.5%)	31 (77.5%)
SF6D, M4	16 (19%)	70 (82%)	12 (30%)	28 (70%)
Total QALDs, Baseline to M4^	17 (20%)	69 (80%)	12 (30%)	28 (70%)
Total Health Service Expenditure, Baseline to Post-Tx~	38 (44%)	48 (56%)	14 (35%)	26 (65%)
Total Health Service Expenditure, Post-Tx to M2~	35 (41%)	51 (59%)	19 (47.5%)	21 (52.5%)
Total Health Service Expenditure, M2 to M4~	41 (47%)	45 (52%)	23 (57.5%)	17 (42.5%)
Total Health System Cost, Baseline to M4~	49 (57%)	37 (43%)	29 (72.5%)	11 (27.5%)
%Productive time, Baseline	4 (5%)	82 (95%)	4 (10%)	36 (90%)
%Productive time, Post-Tx	15 (17%)	71 (83%)	9 (22.5%)	31 (77.5%)
%Productive time, M2	19 (22%)	67 (78%)	12 (30%)	28 (70%)
%Productive time, M4	23 (27%)	63 (73%)	15 (37.5%)	25 (62.5%)
Value of productive time, Baseline to M4#	17 (20%)	69 (80%)	10 (25%)	30 (75%)
PSQI, Baseline	0 (0%)	86 (100%)	0 (0%)	40 (100%)
PSQI, Post-Tx	10 (12%)	76 (88%)	8 (20%)	32 (80%)
PSQI, M2	15 (17%)	71 (83%)	9 (22.5%)	31 (77.5%)
PSQI, M4	16 (19%)	70 (82%)	12 (30%)	28 (70%)
FSS, Baseline	0 (0%)	86 (100%)	0 (0%)	40 (100%)
FSS, Post-Tx	9 (10%)	77 (90%)	7 (17.5%)	33 (82.5%)
FSS, M2	17 (20%)	69 (80%)	9 (22.5%)	31 (77.5%)

FSS, M4	18 (21%)	68 (79%)	12 (30%)	28 (70%)
HADS-A, Baseline	0 (0%)	86 (100%)	0 (0%)	40 (100%)
BPI, Baseline	45 (52%)	41 (47%)	27 (67.5%)	13 (32.5%)

Note. CBT-SF, cognitive behaviour therapy for sleep and fatigue; HE, health education; SD, standard deviation; SF6D, SF36-Based Short-Form Six Dimension Scores; HADS-A, Hospital Anxiety and Depression Scale – Anxiety subscale; BPI, Brief Pain Inventory.

*Excluding treatment group and timepoint where all non-missing.

^Calculated using raw data, missing due to missing SF6D at any time-point or missing duration between time-points.

~ Health System Cost calculated as sum of per patient intervention costs for the CBT-SF or HE interventions and per patient health service expenditure to final follow-up. Health service expenditure to final follow-up calculated as the price-weighted sum of patient-reported use of medications, medical and allied health services and hospitalisations for intervals between timepoints.

Productivity gains/losses to final follow-up calculated using an area-under-the-curve approach, assuming a linear trend between time-points for each patient's percentage time spent in productive activity, applying this percentage to a standard 40 hour working week and multiplying by the average all industries hourly wage rate.

Table S3b: Robustness analysis, including additional covariates in multiple imputation

Outcome	Mean, predicted (SE)		Increment, predicted (95%CI)
	CBT-SF	HE	
Quality of life, QALDs			
Base case[a]	178.70 (2.78)	175.33 (3.67)	3.37 (-4.18, 10.92)
Additional covariates[d]	179.35 (2.56)	175.67 (3.66)	3.68 (-4.13, 11.49)
Total Health System Cost			
Base-case[b]	$6947 (878)	$7428 (1324)	-$481 (-3921, 2960)
Additional covariates[d]	$7215 (976)	$1189 (1277)	-$573 (-3839, 2692)
Productivity Gains / Losses[c]			
Base-case[c]	$48476 (1009)	$47759 (1445)	$717 (-2775, 4208)
Additional covariates[d]	$48585 (1043)	$47720 (1531)	$865 (-2782, 4512)

[a] Quality-Adjusted Life Days (QALDs) calculated based on SF6D Index Scores at each timepoint using an area under the curve approach, assuming a linear trend between time-points for each patient. Marginal means and between-group difference with respect to total QALDs estimated via linear regression on imputed data *adjusting for* duration from Baseline to Post-Treatment follow-up, baseline SF6D scores, and baseline symptomatology as measured by the Hospital Anxiety and Depression Scale. Estimates derived from *mimrgns r.group* and *mimrgns i.group, predict(xb)* after *mi estimate: regress outcome i.group covariates*

[b] Sum of per patient intervention costs for the CBT-SF or HE interventions and per patient health service expenditure to final follow-up. Marginal means and between-group difference with respect to cost-components estimated via linear regression on imputed data *adjusting for* duration from Baseline to Post-Treatment follow-up and baseline symptomatology as measured by the Hospital Anxiety and Depression Scale. Estimates derived from *mimrgns r.group* and *mimrgns i.group, predict(xb)* after *mi estimate: regress outcome i.group covariates*

[c] Per patient productivity gains/losses to final follow-up calculated using an area-under-the-curve approach, assuming a linear trend between time-points for each patient's percentage time spent in productive activity, applying this percentage to a standard 40 hour working week and multiplying by the average all industries hourly wage rate. Marginal means and between-group difference calculated as for health service expenditure and total health system cost but estimated adjusting for additional covariates (baseline productive time and baseline pain).

[d] Multiple imputation as per base-case for each outcome but age, sex, years of education and mode of participation added to the set of covariates in *mi impute chained (regress) imputed = covariates, add(100)*

Supplemental Material S4

Units costs by category of resource use

Category	Description	Unit cost	Source
**Delivery of the CBT-SF / HE Intervention**			
Training & supervision in CBT-SF, therapist	Grade 3, PL1, Psychologist: weekly ordinary full-time wage (effective 12/2023 to 12/2024) of $2,041.50 plus $130.80 qualifications (PhD, DSc) allowance per 38 hour week*	$57.17 per hour	Medical Scientists, Pharmacists & Psychologists Victorian Public Sector (Single Interest Employers) Enterprise Agreement 2021-2025
Training & supervision in CBT-SF, supervisor	Grade 4, PM3, Psychologist: weekly ordinary full-time wage (effective 12/2023 to 12/2024) of $2,501.30 plus $130.80 qualifications (PhD, DSc) allowance per 38 hour week*	$69.27 per hour	Medical Scientists, Pharmacists & Psychologists Victorian Public Sector (Single Interest Employers) Enterprise Agreement 2021-2025
CBT-SF / HE sessions, therapist time	Psychology services hourly rate (effective 06/2023 to 06/2024) for services delivered via telehealth (item 90000T) or in-person (item 90000)	$190.03 per hour	Victorian Transport Accident Commission, Psychology and neuropsychology fees
	MBS item 80010: Psychological therapy health service provided by an eligible clinical psychologist ($166.85 per minimum 50 minute consultation)	$200.22 per hour	Schedule of Medicare Benefits (2024)
CBT-SF / HE sessions, patient time	Non-managerial employees average hourly earnings, all industries (May 2023)	$44.00 / hour	Australian Bureau of Statistics
Handouts and homework sheets	Colour printed 80gsm A4 sheets	$0.25 / side	University of Melbourne Print Room
Telehealth delivery, patient overhead costs	0.25% of weekly internet costs	$76.00 / week	Average weekly internet costs

Category	Description	Unit cost	Source
Face-to-face delivery, patient overhead costs	Zone 1, 2 hour public transport fare	$5.30 / session	Public Transport Victoria
**Health service expenditure**			
Hospital services, base case	Independent Hospital Pricing Authority fee for relevant service provided between 1 July 2023 and 30 June 2024.	NEP*pw= $6,032*pw^	https://www.ihacpa.gov.au/resources/national-efficient-price-determination-2023-24
Hospital services, pessimistic		NEP*1.25*pw^ = $6,032*1.25*pw	
Hospital services, optimistic		NEP*0.75*pw^ = $6,032*0.75*pw	
Physiotherapy, base case	TAC fee for In Rooms Standard Consultation PY602R	$63.64 / service	https://www.tac.vic.gov.au/providers/invoicing-and-fees/fee-schedule/physiotherapy-services-private
Physiotherapy, pessimistic		$63.64*1.25 / service	
Physiotherapy, optimistic		$63.64*0.75 / service	
Occupational therapy, base case	TAC fee for standard consultation by SWEP Green / Amber credentialed OT of up to 30 minutes OTSG02 provided on or after 1 July 2023	$55.51 / service	https://www.tac.vic.gov.au/providers/invoicing-and-fees/fee-schedule/occupational-therapy-services
Occupational therapy, pessimistic	Schedule Fee for MBS item 10958 per minimum 20 minute consultation.	$68.55 / service	https://www9.health.gov.au/mbs/search.cfm
Occupational therapy, optimistic	AHP1 Grade 2, Year 4, OT: weekly ordinary full-time wage (effective from 1 March 2024) of $1,955.60 per 38 hr wk for an hourly rate of $51.46 per hour, or $25.73 per 30 minute service	$25.73 / service	https://www.westernhealth.org.au/Careers/Documents/Awards/Health%20Professionals/Health%20Professionals%20VPS%20Agreement%202021%20-%202026.pdf

Category	Description	Unit cost	Source
Other allied health, base case	TAC hourly rate or service fee for relevant services (e.g. speech pathology, group/individual exercise physiology, dietitian, chiropractor, osteopath, acupuncture, massage, allied health support) provided on or after 1 July 2023, assume 45 minute service for services reimbursed on hourly basis	$30.68 - $91.91 / service	www.tac.vic.gov.au/providers/invoicing-and-fees/fee-schedules
Other allied health, pessimistic	TAC hourly rate or service fee for relevant services (e.g. speech pathology, group/individual exercise physiology, dietitian, chiropractor, osteopath, acupuncture, massage, allied health support) provided on or after 1 July 2023, assume 45 minute service for services reimbursed on hourly basis	$30.68 - $91.91 * 1.25 / service	www.tac.vic.gov.au/providers/invoicing-and-fees/fee-schedules
Other allied health, optimistic	TAC hourly rate or service fee for relevant services (e.g. speech pathology, group/individual exercise physiology, dietitian, chiropractor, osteopath, acupuncture, massage, allied health support) provided on or after 1 July 2023, assume 45 minute service for services reimbursed on hourly basis	$30.68 - $91.91 * 0.75 / service	www.tac.vic.gov.au/providers/invoicing-and-fees/fee-schedules
Psychology, base case	TAC hourly rate of $190.03 for individual clinical psychology services provided on or after 1 July 2023, assume 30 mins per service	$95.02 / hour	https://www.tac.vic.gov.au/providers/invoicing-and-fees/fee-schedule/psychology-services
Psychology, pessimistic	TAC hourly rate of $190.03 for individual clinical psychology services provided on or after 1 July 2023, assume 45 mins per service	$142.52 / service	https://www.tac.vic.gov.au/providers/invoicing-and-fees/fee-schedule/psychology-services
Psychology, optimistic	TAC hourly rate of $49.02 / hour for group clinical psychology services provided on or after 1 July 2023, assume 45 mins per service	$36.77 / service	https://www.tac.vic.gov.au/providers/invoicing-and-fees/fee-schedule/psychology-services
Home care, base case	TAC hourly rate for housekeeping provided on or after 1 July 2023: Housekeeping (per hour) H601, assume duration as specified	$36.46 / hour	https://www.tac.vic.gov.au/providers/invoicing-and-fees/fee-schedule/home-service-non-preferred-providers
Home care, pessimistic	TAC hourly rate for housekeeping provided on or after 1 July 2023: Housekeeping (per hour) H601, assume minimum 2 hours per service	$36.46 / hour	https://www.tac.vic.gov.au/providers/invoicing-and-fees/fee-schedule/home-service-non-preferred-providers
Community services, base case	TAC hourly rate for housekeeping provided on or after 1 July 2023: Housekeeping (per hour) H601	$36.46 / hour	https://www.tac.vic.gov.au/providers/invoicing-and-fees/fee-schedule/home-service-non-preferred-providers
Community services, pessimistic	TAC fee for attendant care (per hour) on or after 16 October 2023: Weekday (Day) 6am to 8pm AT0020	$53.53 / hour	https://www.tac.vic.gov.au/providers/invoicing-and-fees/fee-schedule/attendant-care-nc

Category	Description	Unit cost	Source
Community services, optimistic	Notional full cost per visit of meals-on-wheels	17.70 / service	https://mealsonwheels.com.au/handbook/#:~:text=Cost%20of%20Meals,Care%20subsidy%2C%20are%20as%20follows
Other health services, base case	TAC or MBS Fee for relevant services (e.g. dental, psychiatry) provided on or after 1 July 2023	$111.37- $342.68 / service	www.tac.vic.gov.au/providers/invoicing-and-fees/fee-schedules<br>https://www9.health.gov.au/mbs/search.cfm
Other health services, pessimistic		$111.37- $342.68 * 1.25 / service	
Other health services, optimistic		$111.37- $342.68 * 0.75 / service	
Psychology, base case	TAC hourly rate of $190.03 for individual clinical psychology services provided on or after 1 July 2023, assume 30 mins per service	$95.02 / hour	https://www.tac.vic.gov.au/providers/invoicing-and-fees/fee-schedule/psychology-services
Related MBS medications, base case	Medications for sleep, alertness, mood and pain.	DPMQ for relevant PBS items	Schedule of Pharmaceutical Benefits (2024)
Related MBS medications, pessimistic	Medications for sleep, alertness, mood and pain.	DPMQ*1.25 for relevant PBS items	Schedule of Pharmaceutical Benefits (2024)
Related MBS medications, optimistic	Medications for sleep, alertness, mood and pain.	DPMQ*0.75 for relevant PBS items	Schedule of Pharmaceutical Benefits (2024)
Related OTC medications, base case	Medications for sleep, alertness, mood and pain.	RRP for relevant PBS items	www.chemistwarehouse.com.au

Category	Description	Unit cost	Source
Related OTC medications, pessimistic	Medications for sleep, alertness, mood and pain.	RRP*1.25 for relevant PBS items	
Related OTC medications, optimistic	Medications for sleep, alertness, mood and pain.	RRP*0.75 for relevant PBS items	
**Productivity gains / losses**			
CBT-SF / HE sessions, patient time	Non-managerial employees average hourly earnings, all industries (May 2023)	$44.00 / hour	Australian Bureau of Statistics

* “Ordinary Hourly Rate means 1/38th of the Employee’s weekly wage (including Higher Qualifications allowance which forms part of the Employee’s base salary for all purposes, where there is an entitlement), as prescribed in Schedule 2 of this Agreement” (EBA, p77).

^ Where PW for short-stay outlier calculated as PW=(Short-stay base PW)+LOS*(Short-stay outlier per diem). PW for inlier calculated as PW=Inlier PW. PW for long-stay outlier calculated as PW=(Inlier PW)+(LOS - Inlier Upper Bound)*(Long-stay outlier per diem). NEP is $6,032 per national weighted activity unit 2023–24.

Supplemental Material S5

Results from pessimistic and optimistic sensitivity analyses

Table S5: Incremental cost-effectiveness, pessimistic and optimistic sensitivity

Variable	ΔC	ΔE	ΔC / ΔE	%Acceptable at $200 threshold†	Threshold for 95% confidence*
**Pessimistic sensitivity**					
Clinical outcomes^					
Cost per PSQI[1], Post-Tx	-$54 (-3844, 3736)	**1.78 (0.16, 3.41)**	Tx dominates	61%	$1,954
Cost per PSQI, M2	-$54 (-3844, 3736)	0.46 (-1.17, 2.10)	Tx dominates	54%	Undefined‡
Cost per FSS[2], Post-Tx	-$54 (-3844, 3736)	0.48 (-0.01, 0.97)	Tx dominates	54%	$7,182
Cost per FSS, M2	-$54 (-3844, 3736)	**0.58 (0.09, 1.08)**	Tx dominates	55%	$5,993
Quality of life, QALDs					
Cost per QALD[3]	-$54 (-3844, 3736)	3.37 (-4.18, 10.92)	Tx dominates	67%	Undefined‡
**Optimistic sensitivity**					
Clinical outcomes^					
Cost per PSQI[1], Post-Tx	-$873 (-7781, 6035)	**1.78 (0.16, 3.41)**	Tx dominates	70%	$2,505
Cost per PSQI, M2	-$873 (-7781, 6035)	0.46 (-1.17, 2.10)	Tx dominates	66%	Undefined‡
Cost per FSS[2], Post-Tx	-$873 (-7781, 6035)	0.48 (-0.01, 0.97)	Tx dominates	66%	$9,019
Cost per FSS, M2	-$873 (-7781, 6035)	**0.58 (0.09, 1.08)**	Tx dominates	67%	$7,500
Quality of life, QALDs					
Cost per QALD[3]	-$873 (-7781, 6035)	3.37 (-4.18, 10.92)	Tx dominates	73%	Undefined‡

† Percentage of the joint density of costs and outcomes below the $200 funding threshold where the density is derived via Fieller's method using iprogs.do (8).
* Dollar-value that the (uncertain) funding threshold would have to be in order for 95% of the density to sit below the threshold.
‡ Widest definable confidence interval < 95%.
[1] Cost per point improvement in PSQI scores at the relevant timepoint calculated using base-case estimates of treatment effects for total health system cost to final follow-up and PSQI at the relevant timepoint.
[2] Cost per point improvement in FSS scores at the relevant timepoint calculated using base-case estimates of treatment effects for total health system cost to final follow-up and FSS at the relevant timepoint.
[3] Cost per QALD calculated using base-case estimates of treatment effects for total health system cost and QALDs to final follow-up.
^ Calculated per point improvement (-ΔE) to reflect the fact that relative *reduction* in PSQI and FSS reflects an improvement.

Supplementary References

1. Sittimart M, Rattanavipapong W, Mirelman AJ, Hung TM, Dabak S, Downey LE, et al. An overview of the perspectives used in health economic evaluations. Cost Eff Resour Alloc 2024; 22: 41.
2. Australian_Bureau_of_Statistics. Employee Earnings and Hours, Australia. . 2023 [cited; Available from: https://www.abs.gov.au/statistics/labour/earnings-and-working-conditions/employee-earnings-and-hours-australia/may-2023.
3. Krol M, Brouwer W. How to estimate productivity costs in economic evaluations. Pharmacoeconomics 2014; 32: 335-344.
4. Brouwer WB, Koopmanschap MA, Rutten FF. Productivity costs in cost-effectiveness analysis: numerator or denominator: a further discussion. Health Econ 1997; 6: 511-514.
5. Brouwer WBF, Koopmanschap MA. The friction-cost method. PharmacoEconomics 2005; 23: 105-111.
6. Gabrio A, Plumpton C, Banerjee S, Leurent B. Linear mixed models to handle missing at random data in trial-based economic evaluations. Health Econ 2022; 31: 1276-1287.
7. Pereira RC, Abreu PH, Rodrigues PP, Figueiredo MAT. Imputation of data Missing Not at Random: Artificial generation and benchmark analysis. Expert Systems with Applications 2024; 249: 123654.
8. Glick H, Doshi J, Sonnad S, Polsky D. Economic evaluation in clinical trials. New York: Oxford University Press, 2007.
